# Crystal structure of anhydrous tripotassium citrate from laboratory X-ray powder diffraction data and DFT comparison

**DOI:** 10.1107/S2056989016011506

**Published:** 2016-07-19

**Authors:** Alagappa Rammohan, James A. Kaduk

**Affiliations:** a Atlantic International University, Honolulu, HI, USA; b Illinois Institute of Technology, Chicago, IL, USA

**Keywords:** crystal structure, powder diffraction, density functional theory, citrate, potassium salt

## Abstract

The crystal structure of anhydrous tripotassium citrate, [K_3_(C_6_H_5_O_7_)]_*n*_, has been solved and refined using laboratory X-ray powder diffraction data, and optimized using density functional techniques. The three unique potassium cations are 6-, 8-, and 6-coordinate (all irregular). The [KO_*n*_] coordination polyhedra share edges and corners to form a three-dimensional framework, with channels running parallel to the *c* axis. The only hydrogen bond is an intra­molecular one involving the hy­droxy group and the central carboxyl­ate group, with graph-set motif *S*(5).

## Abstract 2

The crystal structure of anhydrous tripotassium citrate has been solved and refined using laboratory X-ray powder diffraction data, and optimized using density functional techniques.

## Chemical context   

In the course of a systematic study of the crystal structures of group 1 (alkali metal) citrate salts to understand the anion’s conformational flexibility, ionization, coordination tendencies, and hydrogen bonding, we have determined several new crystal structures. Most of the new structures were solved using powder X-ray diffraction data (laboratory and/or synchrotron), but single crystals were used where available. The general trends and conclusions about the 16 new compounds and 12 previously characterized structures are being reported separately (Rammohan & Kaduk, 2016*a* ▸). Five of the new structures, *viz*. NaKHC_6_H_5_O_7_, NaK_2_C_6_H_5_O_7_, Na_3_C_6_H_5_O_7_, NaH_2_C_6_H_5_O_7_, and Na_2_HC_6_H_5_O_7_, have been published recently (Rammohan & Kaduk, 2016*b* ▸,*c* ▸,*d* ▸,*e* ▸; Rammohan *et al.*, 2016 ▸), and two additional structures, *viz*. KH_2_C_6_H_5_O_7_ and KH_2_C_6_H_5_O_7_(H_2_O)_2_, have been communicated to the Cambridge Structural Database (Kaduk & Stern, 2016*a* ▸,*b* ▸).
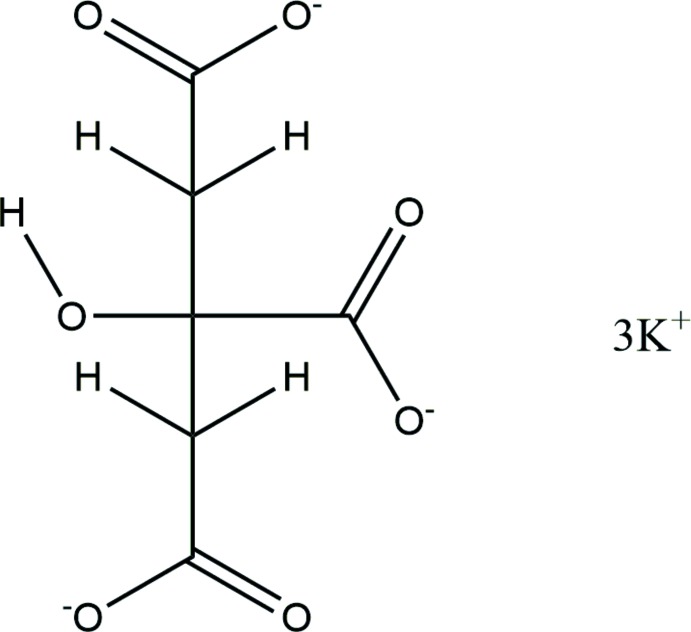


## Structural commentary   

**Figure 1:**
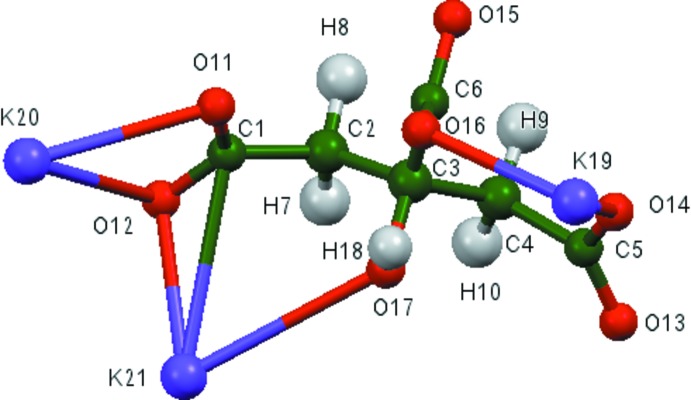
The asymmetric unit of the title compound, showing the atom numbering. Atoms are represented by 50% probability spheroids.

**Figure 2:**
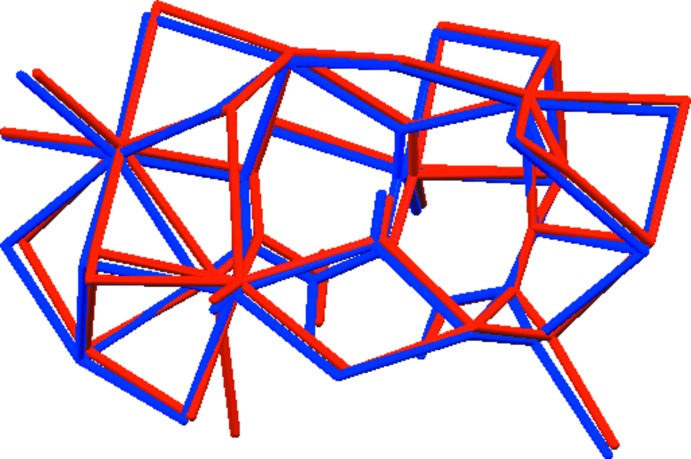
Comparison of the refined and optimized structures of anhydrous tripotassium citrate. The refined structure is in red, and the DFT-optimized structure is in blue.

The asymmetric unit of the title compound is shown in Fig. 1 ▸. The r.m.s. deviation of the non-hydrogen atoms between the Rietveld-refined and the DFT-optimized structures is 0.117 Å (Fig. 2 ▸). The maximum deviation is 0.260 Å, at O14. The good agreement between the two structures is strong evidence that the experimental structure is correct (van de Streek & Neumann, 2014 ▸). This discussion uses the DFT-optimized structure. Most of the bond lengths, bond angles, and torsion angles fall within the normal ranges indicated by a *Mercury Mogul* Geometry Check (Macrae *et al.*, 2008 ▸). Only the C4—C5 bond length [refined = 1.511 (5), optimized = 1.536, *Mogul* average = 1.498 (12) Å, *Z*-score = 3.1], and the C3—C2—C1 [refined = 115 (2), optimized = 115.0, *Mogul* average = 103 (2)°] and O17—C3—C2 angles [refined = 107 (2), optimized = 109.6, Mogul average = 106 (2)°] are flagged as unusual. The citrate anion occurs in the *trans,trans*-conformation, which is one of the two low-energy conformations of an isolated citrate. The central carboxyl­ate group and the hy­droxy group occur in the normal planar arrangement. Both terminal carboxyl­ate groups O11/O12 and O13/O14 chelate to a single potassium cation (K20 for each). The terminal carboxyl­ate oxygen atom O12 and the hy­droxy O17 atom chelate to K21, and the terminal carboxyl­ate oxygen atoms O13 and O17 chelate to K19. The terminal/central pairs O11/O16, O14/O16, O11/O15, and O14/O15 chelate to K21, K19, K19, and K21, respectively. The three potassium cations K19, K20, and K21 are 6-, 8-, and 6-coordinate, respectively (all irregular, using a K—O cut-off distance of 3.24 Å). Their bond-valence sums are 1.12, 1.03, and 1.12 valence units. The metal-oxygen bonding is ionic, based on the cation charges and the Mulliken overlap populations.

Although the lattice parameters of anhydrous tripotassium citrate are in general similar to those of the monohydrate (Carrell *et al.*, 1987 ▸; CSD code ZZZHVI01), consistent with the difference in water content, the powder patterns differ considerably. Visual examination of the structures shows that the arrangements of the citrate anions are very different. A mechanism for the transformation of one phase into the other is not obvious.

The Bravais–Friedel–Donnay–Harker (Bravais, 1866 ▸; Friedel, 1907 ▸; Donnay & Harker, 1937 ▸) morphology suggests that we might expect blocky morphology for anhydrous tripotassium citrate, with {011} as the principal faces. A second-order spherical harmonic texture model was included in the refinement. The texture index was only 1.001, indicating that preferred orientation was not significant for this rotated flat plate specimen.

## Supra­molecular features   

**Figure 3:**
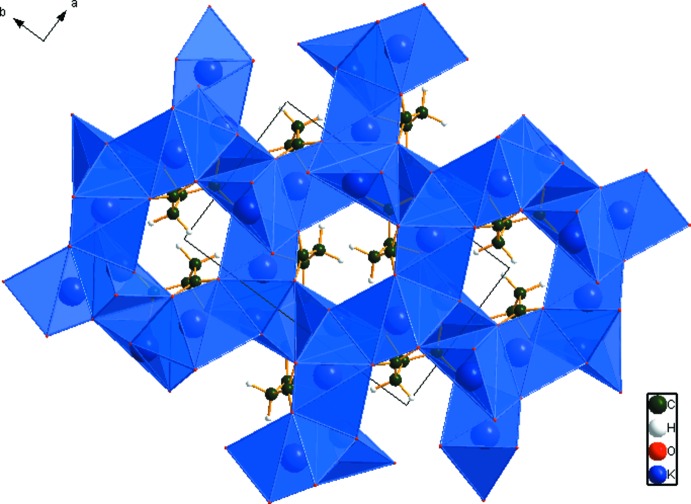
The crystal structure of K_3_C_6_H_5_O_7_, viewed down the *c* axis, with coordination spheres of the potassium cations in polyhedral representation.

The [KO_*n*_] coordination polyhedra share edges and corners to form a three-dimensional framework (Fig. 3 ▸), with channels running down the *c* axis. The only hydrogen bond is an intra­molecular one (Table 1 ▸) involving the hy­droxy group and the central carboxyl­ate group, with graph-set motif *S*(5). The Mulliken overlap population in the hydrogen-acceptor bond is 0.076 *e*. By the correlation in Rammohan & Kaduk (2016*a* ▸), this hydrogen bond accounts for 15.1 kcal per mole of crystal energy.

## Database survey   

Details of the comprehensive literature search for citrate structures are presented in Rammohan & Kaduk (2016*a* ▸). A reduced-cell search of the cell of anhydrous tripotassium citrate in the Cambridge Structural Database (Groom *et al.*, 2016 ▸) (increasing the default tolerance from 1.5 to 2.0%) yielded 208 hits, but limiting the chemistry to C, H, K, and O only resulted in no hits. The powder pattern is now contained in the the Powder Diffraction File (ICDD, 2015 ▸) as entry 00-064-1370.

## Synthesis and crystallization   

Potassium citrate monohydrate was synthesized by dissolving 2.0796 g (10.0 mmole) H_3_C_6_H_5_O_7_(H_2_O) in 20 ml deionized water. 2.0731g K_2_CO_3_ (15.0 mmole, Sigma-Aldrich) was added to the citric acid solution slowly with stirring. The resulting clear colourless solution was evaporated to dryness in a 333 K oven. The powder pattern matched PDF entry 02-064-1651, confirming the structure as potassium citrate monohydrate (Carrell *et al.*, 1987 ▸). The monohydrate was heated at 15 K min^−1^ to 498 K, and held there for two minutes (the white solid started to discolour). The white solid was removed from the oven, and immediately placed in a sealed glass jar to cool.

## Refinement details   

Crystal data, data collection and structure refinement details are summarized in Table 2 ▸. The white solid was ground and blended with NIST SRM 640b Si inter­nal standard in a mortar and pestle. The specimen was protected from the atmosphere by an 8 micron Kapton film attached to the sample holder with petroleum jelly. (The sample hydrates slowly on contact with ambient atmosphere.)

**Figure 4:**
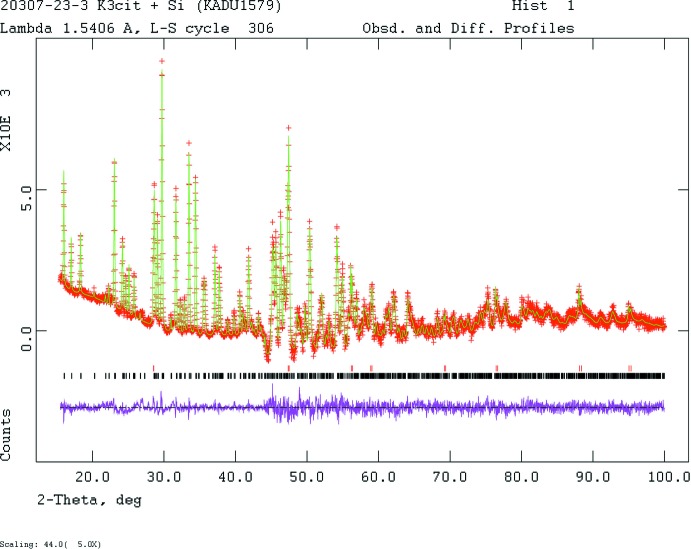
Rietveld plot for the refinement of K_3_C_6_H_5_O_7_. The red crosses represent the observed data points, and the green line is the calculated pattern. The magenta curve is the difference pattern, plotted at the same scale as the other patterns. The vertical scale for angles > 44.0° has been multiplied by a factor of 5. The lower row of black tick marks indicates the reflection positions for the major phase and the upper row of red tick marks is for the silicon inter­nal standard.

The pattern (Fig. 4 ▸) was indexed on a primitive ortho­rhom­bic unit cell using ITO (Visser, 1969 ▸). Manual examination of the systematic absences suggested space group *Pna*2_1_. Pseudo-Voigt profile coefficients were as parameterized in Thompson *et al.* (1987 ▸) with profile coefficients for Simpson’s rule integration of the Pseudo-Voigt function according to Howard (1982 ▸). The asymmetry correction of Finger *et al.* (1994 ▸) was applied and microstrain broadening by Stephens (1999 ▸). The structure was solved with *FOX* (Favre-Nicolin & Černý, 2002 ▸) using a citrate moiety and three potassium atoms as fragments. The structure was refined by the Rietveld method using *GSAS*/*EXPGUI* (Larson & Von Dreele, 2004 ▸; Toby, 2001 ▸). All C—C and C—O bond lengths were restrained, as were all bond angles. The hydrogen atoms were included at fixed positions, which were recalculated during the course of the refinement using *Materials Studio* (Dassault Systemes, 2014 ▸). The *U*_iso_ values of the atoms in the central and outer portions of the citrate anion were constrained to be equal, and the *U*_iso_ values of the hydrogen atoms were constrained to be 1.3× those of the atoms to which they are attached.

The ADDSYM module of *PLATON* (Spek, 2009 ▸) suggested the presence of an additional centre of symmetry, and that the space group was *Pnam*. Refinement in this space group yielded poorer residuals, so we believe that *Pna*2_1_ is the correct space group.

## DFT calculations   

After the Rietveld refinement, a density functional geometry optimization (fixed experimental unit cell) was carried out using *CRYSTAL09* (Dovesi *et al.*, 2005 ▸). The basis sets for the C, H, and O atoms were those of Gatti *et al.* (1994 ▸), and the basis set for K was that of Dovesi *et al.* (1991 ▸). The calculation used 8 *k*-points and the B3LYP functional, and took about 66 h on a 2.4 GHz PC. The *U*_iso_ values from the Rietveld refinement were assigned to the optimized fractional coord­inates.

## Supplementary Material

Crystal structure: contains datablock(s) KADU1578_publ, kadu1578_DFT, KADU1578_overall, KADU1578_phase_1, KADU1578_phase_2, KADU1578_p_01. DOI: 10.1107/S2056989016011506/wm5301sup1.cif

CCDC references: 1493594, 1481346, 1493593

Additional supporting information: crystallographic information; 3D view; checkCIF report

## Figures and Tables

**Table 1:** Hydrogen-bond geometry (Å, °)

*D*—H⋯*A*	*D*—H	H⋯*A*	*D*⋯*A*	*D*—H⋯*A*
O17—H18⋯O16	0.983	1.814	2.552	129.1

**Table 2:** Experimental details

	Phase 1	Phase 2
Crystal data		
Chemical formula	[K_3_(C_6_H_5_O_7_)]	Si
*M*_r_	306.39	28.09
Crystal system, space group	Orthorhombic, *P* *n* *a*2_1_	Cubic, *F* *d* 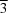 *m*
Temperature (K)	300	300
*a*, *b*, *c* (Å)	7.7062 (2), 12.4693 (3), 10.4241 (2)	5.43105, 5.43105, 5.43105
α, β, γ (°)	90, 90, 90	90, 90, 90
*V* (Å^3^)	1001.66 (3)	160.20
*Z*	4	8
Radiation type	*K*α_1_, *K*α_2_, λ = 1.540629, 1.544451 Å	*K*α_1_, *K*α_2_, λ = 1.540629, 1.544451 Å
Specimen shape, size (mm)	Flat sheet, 24 × 24	Flat sheet, 24 × 24

Data collection		
Diffractometer	Bruker D2 Phaser	Bruker D2 Phaser
Specimen mounting	Normal sample holder with Kapton film	Normal sample holder with Kapton film
Data collection mode	Reflection	Reflection
Scan method	Step	Step
2θ values (°)	2θ_min_ = 4.908, 2θ_max_ = 69.916, 2θ_step_ = 0.020	2θ_min_ = 4.908, 2θ_max_ = 69.916, 2θ_step_ = 0.020

Refinement		
*R* factors and goodness of fit	*R*_p_ = 0.038, *R*_wp_ = 0.049, *R*_exp_ = 0.034, *R*(*F* ^2^) = 0.059, χ^2^ = 2.103	*R*_p_ = 0.038, *R*_wp_ = 0.049, *R*_exp_ = 0.034, *R*(*F* ^2^) = 0.059, χ^2^ = 2.103
No. of parameters	73	73
No. of restraints	29	29

The same symmetry and lattice parameters were used for the DFT calculation. Computer programs: *DIFFRAC.Measurement* (Bruker, 2009 ▸), *FOX* (Favre-Nicolin & Černý, 2002 ▸), *GSAS* (Larson & Von Dreele, 2004 ▸), *EXPGUI* (Toby, 2001 ▸), *DIAMOND* (Crystal Impact, 2015 ▸) and *publCIF* (Westrip, 2010 ▸).

## supplementary crystallographic information

### Crystal data

**Table:**

[K_3_(C_6_H_5_O_7_)]	*c* = 10.4222 (3) Å
*M**_r_* = 306.37	*V* = 1001.66 Å^3^
Orthorhombic, *P**N**A*2_1_	*Z* = 4
*a* = 7.7074 (2) Å	Cu *K*α radiation, λ = 1.5418 Å
*b* = 12.4676 (3) Å	*T* = 300 K

### Data collection

**Table:**

Density functional calculation	*k* = →
*h* = →	*l* = →

### Fractional atomic coordinates and isotropic or equivalent isotropic displacement parameters (Å^2^)

**Table:**

	*x*	*y*	*z*	*U*_iso_*/*U*_eq_	
C1	0.83999	0.87608	0.28090	0.03110*	
C2	0.91941	0.91369	0.40840	0.04090*	
C3	0.80507	0.89495	0.52759	0.04090*	
C4	0.91865	0.91285	0.64734	0.04090*	
C5	0.83786	0.87569	0.77456	0.03110*	
C6	0.64533	0.97237	0.52736	0.03110*	
H7	1.04170	0.87092	0.42230	0.05310*	
H8	0.94795	0.99967	0.40314	0.05310*	
H9	0.94862	0.99862	0.65281	0.05310*	
H10	1.04015	0.86912	0.63386	0.05310*	
O11	0.69006	0.90966	0.25219	0.03110*	
O12	0.93097	0.81435	0.21142	0.03110*	
O13	0.92734	0.81335	0.84433	0.03110*	
O14	0.68837	0.91019	0.80291	0.03110*	
O15	0.67248	1.07226	0.52772	0.03110*	
O16	0.49925	0.92599	0.52665	0.03110*	
O17	0.74146	0.78645	0.52713	0.03110*	
H18	0.61565	0.79949	0.52593	0.04040*	
K19	0.35417	0.87929	0.75053	0.04550*	
K20	0.13670	0.70910	0.02740	0.04550*	
K21	0.14595	0.37878	0.80253	0.04550*	

### Bond lengths (Å)

**Table:**

C1—C2	1.536	C4—C5	1.536
C1—O11	1.265	C4—H9	1.096
C1—O12	1.268	C4—H10	1.093
C2—C3	1.541	C5—O13	1.268
C2—H7	1.093	C5—O14	1.265
C2—H8	1.096	C6—O15	1.263
C3—C4	1.541	C6—O16	1.266
C3—C6	1.564	O17—H18	0.983
C3—O17	1.439		

### Hydrogen-bond geometry (Å, º)

**Table:**

*D*—H···*A*	*D*—H	H···*A*	*D*···*A*	*D*—H···*A*
O17—H18···O16	0.983	1.814	2.552	129.1
